# Hantaviruses in Rodents and Humans, Inner Mongolia Autonomous Region, China

**DOI:** 10.3201/eid1506.081126

**Published:** 2009-06

**Authors:** Yong-Zhen Zhang, Feng-Xian Zhang, Na Gao, Jian-Bo Wang, Zhi-Wei Zhao, Ming-Hui Li, Hua-Xin Chen, Yang Zou, Alexander Plyusnin

**Affiliations:** Chinese Center for Disease Control and Prevention, Changping, Beijing, People’s Republic of China (Y.-Z. Zhang, F.-X. Zhang, N. Gao, M.-H. Li, H.-X. Chen, Y. Zou); Huhehaote Center for Disease Control and Prevention, Huhehaote, Inner Mongolia Autonomous Region, People’s Republic of China (F.-X. Zhang); Yakeshi Center for Disease Control and Prevention,Yakeshi, Inner Mongolia Autonomous Region, People’s Republic of China (J.-B. Wang); Bayannaoer Center for Disease Control and Prevention, Bayannaoer, Inner Mongolia Autonomous Region, People’s Republic of China (Z.-W. Zhao); Haartman Institute, University of Helsinki, Helsinki, Finland (A. Plyusnin)

**Keywords:** Viruses, zoonoses, hemorrhagic fever with renal syndrome, hantavirus, Hantaan virus, Seoul virus, China, Mongolia, research

## Abstract

Surveys were carried out in 2003–2006 to better understand the epidemiology of hantaviruses in the Inner Mongolia Autonomous Region of China (Inner Mongolia). Hemorrhagic fever with renal syndrome (HFRS) was first reported in this region in 1955 and has been an important public health problem here since then. During 1955–2006, 8,309 persons with HFRS were reported in Inner Mongolia (average incidence rate 0.89/100,000), and 261 (3.14%) died. Before the 1990s, all HFRS cases occurred in northeastern Inner Mongolia. Subsequently, HFRS cases were registered in central (1995) and western (1999) Inner Mongolia. In this study, hantaviral antigens were identified in striped field mice (*Apodemus agrarius*) from northeastern Inner Mongolia and in Norway rats (*Rattus norvegicus*) from middle and western Inner Mongolia. Phylogenetic analysis of hantaviral genome sequences suggests that HFRS has been caused mainly by Hantaan virus in northeastern Inner Mongolia and by Seoul virus in central and western Inner Mongolia.

## Abstract 2

Vigilance is needed to prevent hemorrhagic fever renal syndrome caused by Hantaan and Seoul viruses in this region.

Hantaviruses, members of the family *Bunyaviridae* and genus *Hantavirus*, can cause 2 human zoonoses: hemorrhagic fever with renal syndrome (HFRS), seen in Asia and Europe; and hantavirus pulmonary syndrome, seen in the Western Hemisphere (*1*). Rodents are a main virus reservoir and a source of human infection. Transmission of hantaviruses from rodents to humans generally occurs through inhalation of aerosolized excreta (*1**,**2*). In hantavirus-endemic areas, HFRS outbreaks have occurred among farmers and others who have close contact with excreta of infected rodents (*1**–**3*).

HFRS has been recognized as a notable public health problem in China (*4**,**5*). Currently, HFRS is endemic in 28 of 31 provinces in mainland China (*5**,**6*). HFRS cases have occurred mainly in China’s northeastern, eastern, central, and southwestern parts, which are characterized by humid and semihumid zones, but HFRS has rarely occurred in northwestern China, which is in an arid zone (*2**,**4**–**7*). Although 7 sero/genotypes of hantaviruses have been identified in China (*8**–**11*), only Hantaan virus (HTNV), which is carried by striped field mice (*Apodemus agrarius*), and Seoul virus (SEOV), which is carried by Norway rats (*Rattus norvegicus*), are known to cause HFRS in China (*4**,**5**,**8*). The clinical disease caused by HTNV is more severe than that caused by SEOV.

The province of Inner Mongolia in China is located southeast of the Mongolia plateau. It is a frontier area of north China, extending 2,400 km from east to west and 1,700 km from north to south (Figure 1). The first HFRS outbreak was reported in 1955 in the town of Tulihe in the Hulunbeier District (*12*). HFRS cases for the next 40 years mainly occurred in northeastern Inner Mongolia. No cases were reported in the central and western parts until 1995, when outbreaks of HFRS occurred in the Huhehaote District and then in the Bayannaoer District. Previous epidemiologic investigations have suggested the presence of 2 pathogenic hantaviruses in Inner Mongolia: HNTV and SEOV (*8**,**12**–**14*). Recently, we found that Khabarovsk virus (KHAV) is circulating in voles (*Microtus maximowiczii*) in the area surrounding the town of Yakeshi in the Hulunbeier District (*10*). However, it is unknown whether the vole-associated hantaviruses, which include KHAV, are human pathogens.

**Figure 1:**
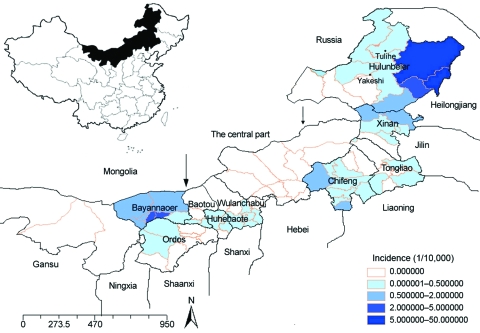
Geographic distribution and average annual incidence of hemorrhagic fever with renal syndrome by district in Inner Mongolia, China, 2001–2006. Arrows mark, from left to right, divisions between western, central, and eastern Inner Mongolia.

In this study, we analyzed hantavirus disease in Inner Mongolia, including death resulting from it and its geographic distribution and dynamics. We also carried out epizootiologic surveys and defined causative agent(s) of hantavirus disease outbreaks in the western parts of the region. Our results suggested that HTNV, associated with striped field mice (*A. agrarius*), was mainly responsible for HFRS cases in northeastern Inner Mongolia; SEOV, associated with Norway rats (*R. norvegicus*), caused outbreaks in the central and western parts of Inner Mongolia.

## Materials and Methods

### Collection of Data for HFRS Cases

Since 1950, HFRS has been a class B notifiable disease in China; thus, annual numbers of human HFRS cases and their distribution have been archived. Records for HFRS cases during 1955–2006 were obtained from the Inner Mongolia Center for Disease Control and Prevention. Before 1982, HFRS cases were defined by a national standard of clinical criteria. As of 1982, cases were also confirmed by detecting antibodies against hantavirus in patients’ serum samples.

### Trapping of Rodents and Screening

From spring 2003 through autumn 2006, rodents were captured in fields and residential areas of 3 HFRS-endemic districts: Hulunbeier, Huhehaote, and Bayannaoer. To capture the rodents, snap-traps were set at 5-m intervals and baited with peanuts. Trapped animals were identified according to previously described criteria (*2**,**4*). Lung tissues from the animals were stored immediately at –196°C and then transported to our laboratory in Beijing for processing. Hantavirus-specific antigens in lungs were detected by indirect immunofluorescent antibody assay as described previously (*15*). Scattered granular fluorescence in the cytoplasm was considered a positive reaction.

### Reverse Transcription–PCR and Sequencing

Total RNA was extracted from the lung tissue samples with the TRIzol reagent (Invitrogen, Beijing, China), according to the manufacturer’s instructions. cDNAs were synthesized with avian myelobalastosis virus reverse transcriptase (Promega Biotech, Beijing, China) in the presence of primer P14 (Table 1) (*16*). Partial small (S) segment sequences (which encode the nucleocapsid protein [N]) of SEOV (nt 584–1019) were amplified with primers HV-SFO and HV-SRO for initial PCR (*17*) and with primers SEO-SF and SEOV-SR (*18*) for the second round of amplification, which yielded the 437-bp product (Table 1). For amplification of HTNV partial S segment sequences (nt 462–1025), primers HV-SFO and HV-SRO (*17*) were used for initial PCR, and primers HSF and HSR (*18*) were used for nested PCR, which amplifies the 564-bp product.

**Table 1:** Specific primers used to detect hantavirus RNA in rodents, Inner Mongolia Autonomous Region, China, 2003–2006*

Type	Primer	Sequence (5′ → 3′)	Segment†	Reference
	P14	TAGTAGTAGACTCC	L, M, S	(*16*)
	HV-SFO	GGCCAGACAGCAGATTGG	S (+)	(*17*)
	HV-SRO	AGCTCAGGATCCATGTCATC	S (–)	(*17*)
HTNV	HSF	AACAAGAGGAAGGCAAACAAC	S (+)	(*18*)
	HSR	GCCCCAAGCTCAGCAATACC	S (–)	(*18*)
SEOV	SEO-SF	TGCCAAACGCCCAATCCA	S (+)	(*18*)
	SEO-SR	GCCATCCCTCCGACAAACAA	S (–)	(*18*)

*HTNV, Hantaan virus; SEOV, Seoul virus. †The hantavirus genome consists of 3 segments defined as small (S), medium (M), and large (L). The segments encode the nucleocapsid protein, 2 envelope glycoproteins, and viral RNA-dependent RNA polymerase, respectively.

The PCR products were gel purified using QIAquick Gel Extraction kit (QIAGEN, Beijing, China), according to the manufacturer’s instructions, and cloned into the pMD18-T vector (TaKaRa Biotechnology, Dalian, China). The ligated products were transformed into JM109 competent cells. DNA sequencing was performed with the ABI-PRISM Dye Termination Sequencing kit and ABI 373-A genetic analyzer (Applied Biosystems, Carlsbad, CA, USA). At least 2 cDNA clones were used to determine each viral sequence. If discrepancies occurred, a third cDNA clone was sequenced.

### Phylogenetic Analysis

**Figure 2:**
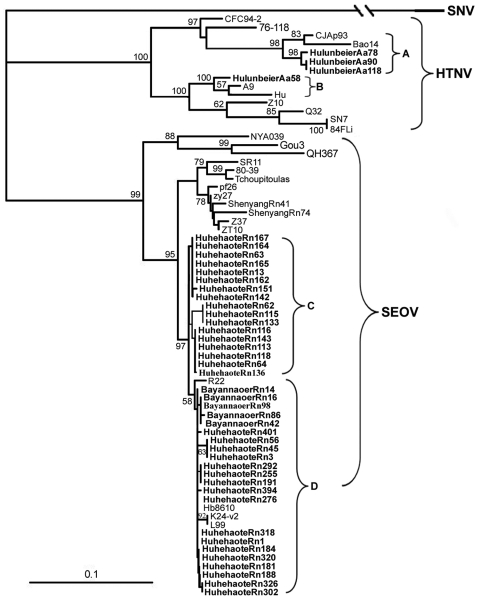
Phylogenetic tree of hantaviruses from rodents in Inner Mongolia, China, 2003–2006. The tree is based on partial sequences of the small (S) segment (nt 620–999 for Seoul virus [SEOV] and nt 614–993 for Hantaan virus [HTNV]). PHYLIP program package (3.65) (http://evolution.genetics.washington.edu/phylip.html) was used to construct the phylogenetic trees by using the neighbor-joining (NJ) and the maximum likelihood (ML) methods with 1,000 bootstrap replicates. The tree constructed by using the ML method had a similar topology as that constructed by using the NJ method (data not shown). Bootstrap values were calculated from 1,000 replicates; only the values >50% are shown at the branch nodes. The sequence of Sin Nombre virus (SNV) was used as the outgroup. Partial S segment sequences recovered from *A. agrarius* field mice trapped in the Hulunbeier District were designated as HulunbeierAa58, HulunbeierAa78, HulunbeierAa90, and HulunbeierAa118; partial S segment sequences from *R. norvegicus* rats trapped in the Huhehaote District were designated as HuhehaoteRn-; and those from *R. norvegicus* rats trapped in the Bayannaoer District were designated as BayannaoerRn14, BayannaoerRn42, BayannaoerRn86, BayannaoerRn98, and BayannaoerRn116. Sequences obtained in this study are shown in **boldface**. All nucleotide sequence data reported here are available in GenBank (accession nos. FJ514504–FJ514546). The GenBank accession nos. of the other partial S segment sequences are SNV/NM H10 (L25748), HTNV/76-118 (M14626), HTNV/CFC94-2 (X95077), HTNV/CJAp93 (EF208953), HTNV/Bao14 (AB127998), HTNV/A9 (AF329390), HTNV/Hu (AB027111), HTNV/Z10 (AF18498), HTNV/Q32 (AB027097), HTNV/SN7 (AF288657), HTNV/84Fli (AY017064); SEOV/NY039 (EF210131), SEOV/Gou3 (AF288651), SEOV/QH367 (DQ081717), SEOV/SR11 (M34881), SEOV/Tchoupitoulas (AF329389), SEOV/IR461(AF329388), SEOV/BJFT01 (DQ519033), SEOV/L99 (AF488708), SEOV/R22 (AF488707), SEOV/Bjhd01 (AY627049), SEOV/K24-V2 (AF288655), SEOV/Z37 (F187082), SEOV/ZT10 (AY766368), and SEOV/Hb8610 (AF288643). Scale bar indicates genetic distance.

PHYLIP (version 3.65) (http://evolution.genetics.washington.edu/phylip.html) was used to construct phylogenetic trees by using the neighbor-joining and maximum-likelihood methods, with 1,000 bootstrap replicates. Alignments were prepared with ClustalW version 1.83 (www.ebi.ac.uk/Tools/clustalw2/index.html). Nucleotide identities were calculated by using the DNAStar program (DNASTAR, Madison, WI, USA). Hantavirus sequences used in the study were retrieved from GenBank (www.ncbi.nlm.nih.gov/Genbank) (Figure 2).

## Results

### Occurrence of HFRS in Inner Mongolia, 1955–2006

No HFRS cases were registered in Inner Mongolia before 1955, when 265 HFRS cases were reported in the town of Tulihe in the Hulunbeier District. From 1955 through 2006, Inner Mongolia had 8,309 reported cases of HFRS, an average incidence rate of 0.89/100,000 population, and 261 deaths caused by HFRS (3.14% of total cases).

**Figure 3:**
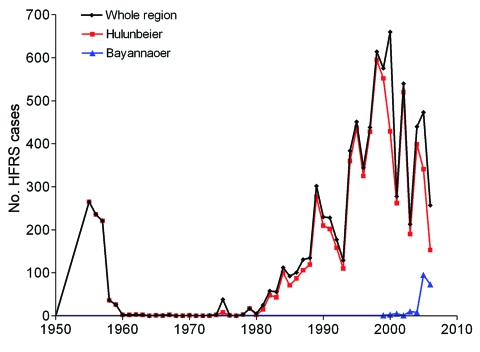
Annual number of cases of hemorrhagic fever with renal syndrome reported by year, Inner Mongolia, China, and selected districts, 1955–2006.

At least 2 major HFRS epidemics have occurred in Inner Mongolia (Figure 3). The first peak was observed during 1955–1957, when 722 cases were registered. After this peak, the number of HFRS cases declined and was relatively stable for the next 24 years, when 168 more cases were registered. However, during this time, the reporting system was suboptimal, so the actual number of HFRS cases might have been higher. As of 1982, the number of cases increased to ≈60 per year, and this ascending phase continued throughout the 1980s. The second epidemic peaked in the 1990s and at the beginning of this century, when 661 HFRS cases, a record number, were reported for 2000.

### Geographic Distribution and Dynamics of HFRS in Inner Mongolia

During 1955–1974, all HFRS cases were registered in the Hulunbeier District (Figure 3). In the late 1970s and in the 1980s, HFRS cases were also found in the neighboring districts of Hulunbeier, Chifeng, Xingan, and Tongliao (Table 2). HFRS cases were later reported in the central part of Inner Mongolia in 1995 (in Huhehaote, the capital of the region) and in the western district of Bayannaoer in 1999). To date, HFRS is prevalent in 9 districts with 56 counties affected (Table 2).

**Table 2:** Number of cases and geographic distribution of hemorrhagic fever with renal syndrome in Inner Mongolia, China, by decade or partial decade, 1955–2006

Years	No. cases	Districts (no. affected counties)
1955–1960	786	Hulunbeier (2)
1961–1970	12	Hulunbeier (5), Chifeng (1)
1971–1980	67	Hulunbeier (7), Chifeng (1), Xingan (1), Tongliao (1)
1981–1990	1,242	Hulunbeier (9), Chifeng (2), Xingan (2), Tongliao (1)
1991–2000	4,002	Hulunbeier (9), Chifeng (6), Xingan (5), Tongliao (4), Huhehaote (3), Baotou (1), Bayannaoer (2), Wulanchabu (2)
2001–2006	2,197	Hulunbeier (9), Chifeng (10), Xingan (6), Tongliao (4), Huhehaote (9), Baotou (4), Bayannaoer (7), Wulanchabu (6), Ordos (1)

Although HFRS cases have been registered in 9 districts, most cases have been reported in the Hulunbeier District since the first outbreak, which occurred in Tulihe in 1955 (Figures 1 and 3). During 1955–2006, a total of 7,367 HFRS cases were reported in the Hulunbeier District, representing 88.7% of all cases registered in Inner Mongolia. Most of these cases (72.2%) occurred during the months of October–January.

In the Huhehaote District, the first HFRS case was reported in 1995. Later, HFRS occurred in the neighboring districts of Baotou and Wulanchabu. The HFRS incidence in these districts has been lower than in the districts of Bayannaoer, Chifeng, Hulunbeier, and Xingan, each of which reported fewer than 10 HFRS cases annually.

In the Bayannaoer District, no HFRS cases were reported before 1999; during 1999–2004, fewer than 10 cases were registered annually. Outbreaks of HFRS occurred in this district in 2005 and 2006, with 95 and 75 cases, respectively, being reported for these years, although this district initiated comprehensive control measures, including vaccination, after the first case occurred. Of 196 cases reported during 1999–2006, 3 people died. In contrast to the HFRS cases in the Hulunbeier District, more than 60% of cases in the Bayannaoer District occurred during March–June.

### Screening of Rodents for Hantaviral Antigens in Lung Tissues

From spring 2003 through autumn 2006, 1,466 rodents belonging to 11 species were trapped in the fields and residential areas of Inner Mongolia (Table 3). Of that total, 529 were trapped in the Hulunbeier District, 15 (13 *A. agrarius* and 2 *A*. *peninsulae* field mice) of which were found by indirect immunofluorescent antibody assay to be positive for hantavirus antigens. Of the 594 rodents trapped in the Huhehaote District, 38 *R. norvegicus* rats were positive. In the Bayannaoer District, 343 rodents were trapped, among which 8 *R. norvegicus* rats and 8 *Meriones meridianus* gerbils were found to be positive. The identification of hantavirus antigens in *M. meridianus* gerbils suggests that this rodent species is a new carrier and may carry additional unidentified hantavirus(es).

**Table 3:** Prevalence of hantavirus(es) in rodents by species, district, and ecological location, Inner Mongolia, China, 2003–2006*

Species	Hulunbeier District			Huhehaote District			Bayannaoer District	
	Field/ grassland	Residential area		Field/desert grassland	Residential area		Field/desert grassland	Residential area
*Apodemus agrarius*	132/9/3	81/4/1		0/0/0	0/0/0		0/0/0	0/0/0
*Apodemus peninsulae*	25/2/0	11/0/0		0/0/0	0/0/0		0/0/0	0/0/0
*Rattus norvegicus*	12/0/0	23/0/0		79/5/4	418/33/30		0/0/0	132/8/5
*Mus musculus*	0/0/0	7/0/0		0/0/0	21/0/0		0/0/0	19/0/0
*Microtus maximowiczii*	38/3/3	26/2/2		0/0/0	0/0/0		0/0/0	0/0/0
*Myodes rufocanus*	18/0/0	3/0/0		0/0/0	0/0/0		0/0/0	0/0/0
*Myodes rutilus*	89/0/0	54/0/0		0/0/0	0/0/0		0/0/0	0/0/0
*Cricetulus barabensis*	7/0/0	3/0/0		33/0/0	0/0/0		41/0/0	0/0/0
*Meriones meridianus*	0/0/0	0/0/0		43/0/0	0/0/0		140/8/0	0/0/0
*Allactage sibirica*	0/0/0	0/0/0		0/0/0	0/0/0		6/0/0	0/0/0
*Dipus sagitta*	0/0/0	0/0/0		0/0/0	0/0/0		5/0/0	0/0/0
Total	321/14/6	208/6//3		155/5/4	439/33/30		192/8/0	151/8/5

*Data show no. of rodents captured/no. positive for hantavirus antigen/no. PCR positive.

### Phylogenetic Analyses

To establish molecular epidemiologic links between hantaviruses in rodents and HFRS outbreaks in central, western, and northeastern Inner Mongolia, we recovered partial S segment sequences (nt 463–1025 for HTNV; nt 584–1019 for SEOV) from the rodent tissue samples and subjected them to genetic analysis. Four of 13 hantaviral antigen–positive mice (*A. agrarius*) from the Hulunbeiere District, 34 of 38 hantaviral antigen–positive rats (*R. norvegicus)* from the Huhehaote District, and 5 of 8 hantaviral antigen–positive rats (*R. norvegicus)* from the Bayannaoer District were found positive by reverse transcription–PCR. The hantaviral sequences recovered from these rodents were designated respectively as HulunbeierAa-, HuhehaoteRn-, and BayannaoerRn- (Figure 2). Unfortunately, our attempts to amplify partial S segment sequences from antigen-positive *M. meriidanus* gerbils were unsuccessful. In addition, KHAV–specific partial S segment sequences were amplified earlier from 5 voles of species *M. maximowiczii* (*10*).

As expected, partial S segment sequences recovered from *A. agrarius* mice were more closely related to HTNV (sequence identities 79.2%–98.9%) than to other known hantaviruses. On the phylogenetic tree, 4 strains recovered from *A. agrarius* mice belonged to 2 lineages (A and B) (Figure 2). The sequences HulunbeierAa78, HulunbeierAa90, and HulunbeierAa118 showed a closer evolutionary relationship to strain Bao14 isolated from *A.* *agrarius* mice trapped in the neighboring province of Heilongjiang (*8*) and to strain CJAp93 isolated from *A.* *peninsulae* mice captured in Jilin Province (*19*). The sequence HulunbeierAa58 clustered together with strain A9 isolated from *A. agrarius* mice from Jiangsu Province and strain Hu isolated from a person in the Hubei Province (*8*).

All sequences recovered from *R. norvegicus* rats showed higher identity to SEOV (80.2%–99.5%) than to HTNV or other hantavirus types. These sequences were closely related to each other, with 92.5%–99.9% sequence identity. The partial S sequences from *R. norvegicus* rats from the Huhehaote District formed 2 clusters (marked C and D, Figure 2). Although the C cluster included only sequences from the Huhehaote District, the D cluster included sequences from both the Huhehaote and Bayannaoer Districts and also included sequences from the Chinese strains K24 (from Zhejiang Province), Hb8610 (from Shanxi Province), L99 (from Jiangxi Province), and R22 (from Henan Province) (*8*). All sequences recovered from *R. norvegicus* rats from the Bayannaoer District belonged to cluster D, suggesting that SEOV variants causing the HFRS outbreak in the Bayannaoer District are genetically very close to those from the Huhehaote District.

## Discussion

In this study, we describe the incidence, geographic distribution, and dynamics of HFRS in Inner Mongolia from 1955 through 2006. HFRS had been a serious concern in the region for the past 20 years. Habitat differences, host distribution, rodent serosurveys, and phylogenetic analysis suggest that HFRS in the northeastern region has been caused mainly by HTNV, and the HFRS outbreaks occurring in the central and western parts have been caused mainly by SEOV.

The occurrence and epidemics of HFRS are influenced by both natural (e.g., ecological) and occupational factors (*2**,**3**,**7**,**20**,**21*). Many hantavirus infections have occurred in persons of low socioeconomic status because of poor housing conditions (*1**,**2**,**5*). In China, the highest HFRS incidence occurred in the humid and semihumid areas, where annual precipitation levels are 400–800 mm, and no cases have been reported from the arid areas, where the precipitation is <200 mm (*6**,**7*). Notably, most HFRS cases occurred in rural areas (*5**,**7*). The Hulunbeier District is situated in northeast Inner Mongolia and belongs to humid areas. The largest coniferous forest in north China is situated there. Consequently, the *A. agrarius* mouse is a species that most frequently carries the hantavirus antigen in this area (Table 3), a finding consistent with earlier epidemiologic investigations (*12**–**14*). On the other hand, housing conditions for most farmers in the Hulunbeier District are poorer than in neighboring areas because of the relatively slow development of the local economy in this district. Thus, the local natural conditions, which support a high density of rodents and a high prevalence of hantavirus(es), as well as the generally low socioeconomic status of the Hulunbeier District, could contribute to the high numbers of HFRS cases in this district.

Huhehaote and Bayannaoer Districts are located in the central and western parts of Inner Mongolia, respectively. Both belong to a semi-arid or arid zone. Until the 1990s, epizootiologic surveys had not shown the presence of hantaviruses circulating in rodents and infecting humans in these districts (*14*). The first HFRS cases were reported in the Huhehaote District in 1995 and in the Bayannaoer District in 1999. In the Bayannaoer District, a relatively large HFRS outbreak occurred in 2005 and 2006, with 95 and 75 cases reported, respectively. The ecology of central and western Inner Mongolia differs from that of the northeastern part. In the central and western parts, *A. agrarius* mice are absent, and *R. norvegicus* rats are abundant. Consequently, a high number of human hantavirus infections are registered. Since the 1980s, the incidence of HFRS has been high in the provinces of Hebei and Shanxi (*2**,**5**,**6**,**22**–**24*), which share borders with central Inner Mongolia (Figure 3). Particularly, the annual number of HFRS cases reported in Hebei Province was more than 4,000 during 1990–2002 (*6*). *R. norvegicus* rats were the predominant reservoir; hence, SEOV was prevalent in the provinces of Hebei and Shanxi (*2**,**5**,**22**–**24*).

Our phylogenetic analysis showed that SEOV sequences isolated from the rats trapped in Huhehaote and Bayannaoer Districts were closely related to those of strains HB8610 and K24 from Shanxi and Zhejiang Provinces, respectively, and also to sequences of 2 other strains from China (Figure 2). This genetic lineage of SEOV seems to be widely distributed in China (*8**,**15**,**18**,**25**,**26*). Norway rats, the carriers of SEOV, are more invasive than the hosts of other hantaviruses and have dispersed throughout much of the world through various modes of transportation. As a result, SEOV is the only cosmopolitan hantavirus known so far (*27*). Genetic variants of SEOV currently circulating in rats in central Inner Mongolia could come from the provinces of Shanxi or Hebei and then spread westward into the Bayannaoer District due to increased transportation of goods and human migration that followed the rapid economic development in China over the past decades.

Of host species, *A. agrarius* mice carried hantavirus antigens most frequently in the Hulunbeier District, whereas *R. norvegicus* rats were the predominant carriers in the central and western parts of Inner Mongolia (Table 3), where *A. agrarius* mice have never been found because of this species’ desert ecosystem (*14**,**28**,**29*). Phylogenetic analysis confirmed the presence of HTNV in *A. agrarius* mice in the Hulunbeier District and SEOV in *R. norvegicus* rats in the Huhehaote and Bayannaoer Districts. In addition, the peak of HFRS associated with *A. agrarius* mice occurred in the winter, whereas HFRS associated with *R. norvegicus* rats occurred mainly in the spring (*2**,**4*). Thus, our results showed that HFRS in the northeastern part was caused mainly by HTNV, although we demonstrated that KHAV is also circulating in *M*. *maximowiczii* voles in the Hulunbeier District (*10*), and SEOV was responsible for the HFRS outbreaks in the Huhehaote and Bayannaoer Districts.

The annual incidence of HFRS has gradually decreased in China during the past 7 years (*5*). Our data suggest that the HFRS outbreak in the central and western parts of Inner Mongolia was probably caused by SEOV variants. HFRS has occurred in the areas such as the Bayannaoer District, where the disease was not previously reported, even during the 1980s and 1990s when the overall incidence was high (*5*). Whether these newly appearing HFRS cases result from improved surveillance or reflect a recent spread of the virus to these areas remains to be seen. This study reinforces the need for vigilance in preventing HFRS caused by HTNV and SEOV (and perhaps other hantaviruses) in China. This vigilance should include regular surveillance of local rodent populations for evidence of hantavirus infection.
